# Therapeutic and pharmaco-biological, dose-ranging multicentre trial to determine the optimal dose of TRAnexamic acid to reduce blood loss in haemorrhagic CESarean delivery (TRACES): study protocol for a randomised, double-blind, placebo-controlled trial

**DOI:** 10.1186/s13063-017-2420-7

**Published:** 2018-03-01

**Authors:** Anne-Sophie Bouthors, Benjamin Hennart, Emmanuelle Jeanpierre, Anne-Sophie Baptiste, Imen Saidi, Elodie Simon, Damien Lannoy, Alain Duhamel, Delphine Allorge, Sophie Susen

**Affiliations:** 1 CHU Lille, Pole anesthésie réanimation, maternité Jeanne de Flandre, 59000 Lille, France; 2 CHU Lille, Unité fonctionnelle de toxicologie, 59000 Lille, France; 3 CHU Lille, Unité fonctionnelle d’hémostase-hémobiologie, centre biologie pathologie, 59000 Lille, France; 4 CHU Lille, Pharmacie centrale, 59000 Lille, France; 5 Université Lille EA 2604 Unité de biostatistiques, 59000 Lille, France; 6 Université Lille, EA 4483 – IMPECS – IMPact de l’Environnement Chimique sur la Santé humaine, 59000 Lille, France; 7 Université Lille EA2693, 59000 Lille, France; 8 Pole anesthésie-réanimation, maternité Jeanne de Flandre, academic hospital, Avenue Oscar Lambret, 59037 Lille, France

**Keywords:** Postpartum haemorrhage, Caesarean section, Fibrinolysis, Tranexamic acid, Pharmacokinetics, Plasmin, D-dimers

## Abstract

**Background:**

Postpartum haemorrhage (PPH) is the leading cause of maternal death worldwide. Tranexamic acid (TA), an antifibrinolytic drug, reduces bleeding and transfusion need in major surgery and trauma. In ongoing PPH following vaginal delivery, a high dose of TA decreases PPH volume and duration, as well as maternal morbidity, while early fibrinolysis is inhibited. In a large international trial, a TA single dose reduced mortality due to bleeding but not the hysterectomy rate. TA therapeutic dosages vary from 2.5 to 100 mg/kg and seizures, visual disturbances and nausea are observed with the highest dosages. TA efficiency and optimal dosage in haemorrhagic caesarean section (CS) has not been yet determined. We hypothesise large variations in fibrinolytic activity during haemorrhagic caesarean section needing targeted TA doses for clinical and biological efficacy.

**Methods/design:**

The current study proposal is a blinded, randomised controlled trial with the primary objective of determining superiority of either 1 g of TXA or 0.5 g of TXA, in comparison to placebo, in terms of 30% blood-loss reduction at 6 h after non-emergency haemorrhagic caesarean delivery (active PPH > 800 mL) and to correlate this clinical effect in a pharmacokinetics model with fibrinolysis inhibition measured by an innovative direct plasmin measurement regarding plasmatic TA concentration.

A sample size of 342 subjects (114 per group) was calculated, based on the expected difference of 30% reduction of blood loss between the placebo group and the low-dose group, out of which 144 patients will be included blindly in the pharmaco-biological substudy. A non-haemorrhagic reference group will include 48 patients in order to give a reference for peak plasmin level.

**Discussion:**

TRACES trial is expected to give the first pharmacokinetics data to determinate the optimal dose of tranexamic acid to reduce blood loss and inhibit fibrinolysis in hemorrhagic cesarean section.

**Trial registration:**

ClinicalTrials.gov, ID: NCT02797119. Registered on 13 June 2016.

**Electronic supplementary material:**

The online version of this article (doi:10.1186/s13063-017-2420-7) contains supplementary material, which is available to authorized users.

## Background and study rationale

**Fig. 1:**
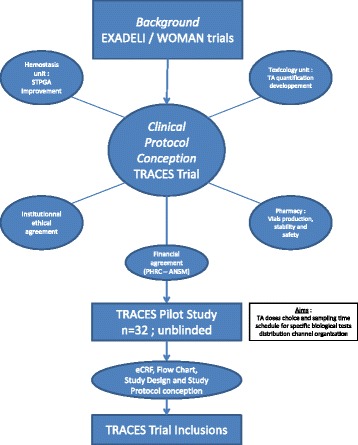
Global view of the TRACES trial conception

This manuscript describes the background and design of the TRACES trial, with a special attention to the updated knowledge on PPH-related coagulopathy (Fig. 1).

### The treatment of the postpartum haemorrhage-induced coagulopathy could contribute to the reduction of maternal morbidity

Postpartum haemorrhage (PPH) remains the leading cause of maternal mortality worldwide [1]. PPH mostly occurs during caesarean section (CS) due to atony and abnormal placental insertion. Haemorrhagic CS represents more than half of severe PPH. PPH-induced coagulopathy can be potentially major and contributes to the severity of PPH [2]. Coagulopathy has been observed as a major component of maternal morbidity after vaginal delivery due to uterine atony [3, 4], but also in patients experiencing intensive care admission after massive PPH [5, 6]. Early hyper-fibrinolysis can be observed at the onset of PPH and becomes maximal 2 h after the beginning of bleeding when no prohaemostatic treatment has been given [7].

Fibrinogen decrease is a central component of PPH-induced coagulopathy concomitant to D-dimer increase and factor II decrease [3, 4, 7]. Active fibrinogenolysis remains a major component of amniotic fluid embolism, fœtal death or abruptio placentae [8, 9]. Tranexamic acid (TA) (Exacyl® Sanofi, Paris, France) reduces D-dimers and the plasmin-antiplasmin (PAP) complex peak observed at PPH onset [7], as well as in abruptio placentae [10].

### Tranexamic acid reduces blood loss and transfusion need

TA reduces bleeding and transfusion need in medical and gynaecological haemorrhage, major surgery and trauma, without major side effects [11, 12].

In obstetric settings, available TA therapeutic efficiency and safety data are limited. Eight placebo-controlled, blinded, randomised trials demonstrated that the prophylactic use of TA reduced postoperative bleeding in elective non-haemorrhagic CS or after vaginal delivery [13–20]. In antepartum anaemic patients, this mild reduction was associated with a decrease in transfusion need [20].

**Fig. 2:**
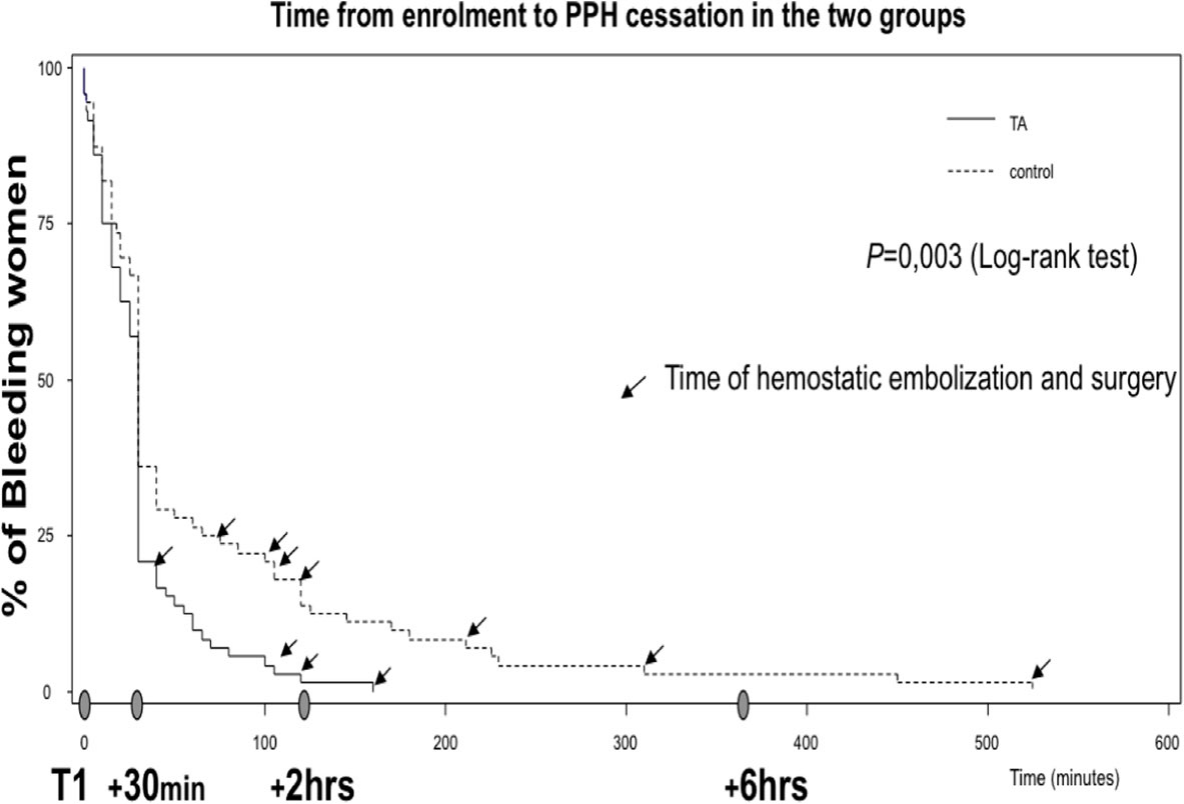
Reduction of postpartum haemorrhage duration in the high-dose-tranexamic-acid-treated group vs the untreated control group

**Fig. 3:**
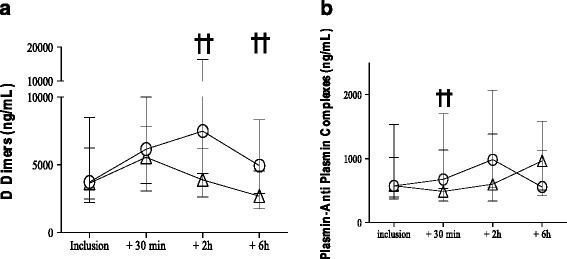
Inhibition of the natural hyperfibrinolysis by a high dose of tranexamic acid administered at the early stage of postpartum haemorrhage. ††*p* < 0.05. Panel **a** (circles) : hemorrhagic non treated group, Panel **b** (triangles): hemorrhagic TA treated group

Only one randomised controlled study (EXADELI trial) tested TA efficiency in active PPH following vaginal delivery [21]. A high dose of 4 g was administered, followed by a continuous infusion of 1 g/h. The EXADELI trial demonstrated a mild, but significant, reduction of the bleeding volume (173 mL vs 221 mL in the control group) and a significant reduction of the delay for PPH cessation measured as a blood flow of less than 50 mL per 10 min (Fig. 2) [21]. Although limited, the bleeding volume reduction was associated with a reduction of the percentage of patients developing severe morbidity, such as anaemia or haemorrhagic shock [21]. Mild side effects were observed, including nausea and visual disturbances, but no acute kidney injury nor seizure or thromboembolism were observed. Renal function was similar or even better in the TA group. Deep vein thrombosis complicated catheter insertion in two patients in the control group and three in the TA group [21]. The major point of the EXADELI trial was the strict measurement of PPH bleeding on special graduated bags and the strict respect to the French timed PPH management [22]. Concomitant administration of prohaemostatic treatments was avoided, except for untraceable PPH, enabling the observation of the natural course of PPH-induced coagulopathy. TA clinical efficiency to reduce bleeding in the haemorrhagic caesarean context has not been previously published. In the only (unpublished) Tunisian randomised, placebo-controlled trial in haemorrhagic CS, TA administration induced a reduction of blood loss and transfusion need (*p* = 0.03). Because the absence of academic literature on CS and the low level of evidence after vaginal delivery, current 2014 French guidelines for PPH management restricted the use of TA to late administration in massive haemorrhage: ‘Tranexamic acid may be of interest in PPH treatment, even if its clinical interest has not been demonstrated in an obstetrics setting. Every clinician is free to use TA. If used, the expert group suggests the administration of a 1-g dose only if PPH resists prostaglandins’ [22]. The WOMAN trial aimed to assess the impact of the early administration of TA on death and hysterectomy in women experiencing PPH [23, 24]. Between 2010 and 2016, 20,060 women with a clinical diagnosis of PPH in 21 low- and middle-income countries were included in this randomised, double-blind, placebo-controlled trial to intravenously receive either 1 g TA (*n* = 10,051) or placebo (*n* = 10,009) in addition to standard care. The WOMAN trial found that TA decreased death due to bleeding (155 (1.5%) vs 191 (1.9%), RR 0.81; 95% CI 0.65–1.00; *p* = 0.045), especially in women given treatment within 3 h of giving birth (89 (1.2%) vs 127 (1.7%), RR 0.69; 95% CI 0.52–0.91; *p* = 0.008). Laparotomy for bleeding was also reduced (82 (0.8%) vs 127 (1.3%), RR 0.64; 95% CI 0.49–0.85; *p* = 0.002) after CS and vaginal delivery. The original composite primary endpoint was not reduced (534 (5.3%) vs 546 (5.5%), RR 0.97; 95% CI 0.87–1.09; *p* = 0.65). Adverse events were not increased in the TA group vs placebo: thromboembolic events (30 (0.3%) vs 34 (0.3%)), seizures (33 (0.3%) vs 43 (0.4%)), and renal failure (129 (1.3%) vs 120 (1.2%)). One could suggest that the TA benefit on mortality may be higher in women with severe PPH, then aim to reserve TA for the more severe situations. However, the data showed that the delayed administration of TA was associated with potential harm. Thus TA, if used, should be given early and empirically as soon as a PPH is diagnosed, and before severe haemorrhage. As the study was based on robust clinical endpoints, neither blood loss estimation nor haemoglobin drop were available. There was no subgroup analysis of TA efficacy according to the volume of bleeding. Laboratory results were not included in the primary analysis but were assessed in a few centres as a part of a secondary analysis [25]. The biological effect of the high TA dose administered in the EXADELI trial was observed and compared to a non-treated haemorrhagic group and a non-haemorrhagic postpartum group. Significant excessive fibrinolysis was noted in the untreated haemorrhagic group vs the non-haemorrhagic group at PPH onset. This excessive fibrinolysis was inhibited in the TA-treated group 30 min and 2 h after TA administration, as demonstrated by the inhibition of the D-dimers and PAP increases (Fig. 3) [7].

Few other cases or case series have previously suggested the efficiency of TA to reduce maternal mortality and morbidity in placental abruption, third trimester placental bleeding or amniotic fluid embolism [10, 26].

The TRACES clinical and pharmacokinetics dose-ranging study objective will be a better understanding of the mechanisms of TA efficacy and the optimal selection of the TA dose.

### A dose-ranging study is needed for TA optimal dose and therapeutic timing assessment

The minimal effective dose of TA is not known. In major cardiac surgery, the 4-g dose followed by a continuous infusion of 1 g/h is the known efficient therapeutic dosage. However, in trauma, as in major surgery, fibrinolysis is an expected part of coagulopathy. In PPH associated with CS, although fibrinolysis appears early and is constant, its intensity is variable and most of the PPH stops after the first step of management. We hypothesis that a 0.5-g dose is sufficient to inhibit the most common fibrinolytic activity in haemorrhagic CS, while some of the CS-associated fibrinolysis is so intense that neither 0.5 g nor 1 g will be enough the inhibit the coagulopathic process.

### The choice of the different dose regimens relevant for study in the haemorrhagic caesarean section context

The higher dose of 4 g and continuous infusion was not selected because of the minor side effects observed in the EXADELI trial [21]. The TA 2-g dosage inhibited fibrinolysis after the 30th min in the treated patients in the EXADELI trial. The dose of 1 g or 10 mg/kg is commonly used prophylactically before CS, but has not yet been tested as a treatment in an actively bleeding haemorrhagic CS. Because of the lack of data on lower doses and TA pharmacokinetics, a low 0.5-g dose should also be tested.

### Study aim and originality

The aim of the study is to determine, out of two doses (a standard and a low dose) compared to placebo, the optimal and minimal dose of an intravenously administered single bolus of TA to reduce blood loss when administered during haemorrhagic CS.

The originality of the trial can be described as follows: no previous study has been published evaluating the clinical impact of TA on blood loss and transfusion need reduction during haemorrhagic CS. The determination of the optimal dose to obtain this clinical impact is of interest for clinicians in the current obstetric practice. TA concentration and innovative direct plasmin peak assessment by a simultaneous generation thrombin plasmin assay (SGTPA) are expected to provide scientific and objective data to determine the optimal dose. The dose-ranging study with a low dose is expected to describe the minimal dose able to inhibit fibrinolysis and to contribute to bleeding reduction while minimising side effects. The first pharmaco-biological observational results allows the choice of this dose ranging. No previous study has tested the low dose in the obstetric literature in either trauma-induced massive haemorrhage or major surgery.

## Methods/design

### Experimental plan

**Fig. 4:**
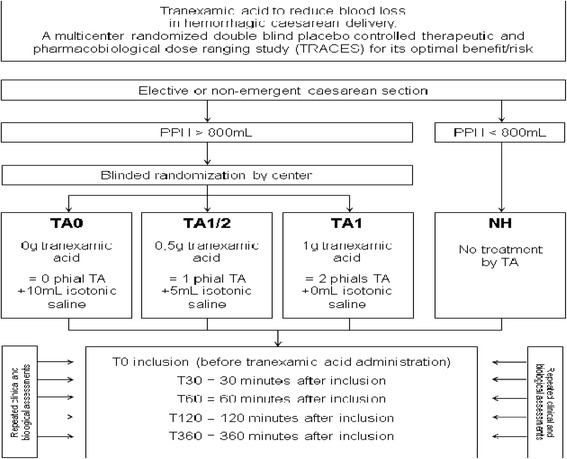
Study design

The TRACES study is a multicentre, randomised, double-blinded, placebo-controlled therapeutic and pharmaco-biological dose-ranging study to evaluate the clinical efficiency of TA on blood-loss reduction in patients experiencing PPH during elective or non-emergency CS (Fig. 4).

### Expert authorities and ethics committee approval

The TRACES trial obtained approval from the competent national authorities (ANSM 201500249926) and the Ethics Committee (CPP 15/50 020216) before beginning the study, in accordance with article L1121-4 of the Public Healthcare Code. This trial has been declared on the clinical trials registration under the number CT 02797119. Registration will be performed in accordance with decree dated 14 November 2006 about gathering data in the national register of individuals participating in biomedical research.

### Participating centres involved in the research programme

The five medical centres are known for their strict multidisciplinary management of PPH, including systematic measurement of blood loss, and for their ability to perform research programmes, such as the EXADELI trial. Anaesthetic and obstetric staff of each centre agreed to their participation to this study and were involved in writing the French guidelines for PPH management [22]. The study will be sponsored by The University and Regional Hospital Centre of Lille, France.

### Study population

The inclusion criteria for the experimental haemorrhagic group concern each patient experiencing a bleeding volume of more than or equal to 800 mL due to surgery or to uterine atony during an elective or non-emergency CS. This experimental group will receive a low dose or a standard dose of treatment (treatment groups) or placebo (control group). The non-haemorrhagic group, where patients experience a bleeding volume of less than 800 mL, is a reference group for biology and will be sampled to obtain a reference profile for the direct plasmin generation test in CS. All patients receive complete information, give their written consent and are covered by social security.

Non-inclusion criteria are as follows: hypersensitivity to the product or its excipient, previous or ongoing arterial or venous thrombosis, disseminated intravascular coagulopathy, except disseminated intravascular coagulopathy (DIC) associated with a predominant fibrinolytic profile, renal failure, previous seizures, severe HELLP syndrome, emergency CS, administration of TA before inclusion, inherited haemorrhagic diseases and low-molecular-weight-heparin administration within 24 h before inclusion, previous inclusion in an interventional trial for 2 months, or patient being unable to consent.

### Study protocol

#### Screening phase – information to subjects

During the routine third-trimester preanaesthestic assessment, every patient is informed about research programmes focussing on PPH and TA in haemorrhagic CS. During the preanaesthestic visit, inclusion and non-inclusion criteria are screened for; full detailed information will be given. Informed consent is signed before caesarean onset.

#### Randomisation, blinding and treatment administration – blind production of TA doses and their concentration and stability assessment

##### Description of the unit dose, packaging and labelling of the study drug

The doses will be packaged blindly for the investigators by the pharmacy of the sponsor. Each package will be numbered following the randomisation table established by centre. The package will include 10-mL syringes or flacons of the studied product. The preparation of the product in each group will be TA1 (10 mL = 1 g TA + 0 mL saline), TA½ (5 mL = 0.5 g TA + 5 mL saline) or TA0 (0 mL = 0 g TA + 10 mL saline). The master randomisation list is furnished to the pharmacy production unit to identify the packages by identification numbers containing information on the centre, on the haemorrhagic or non-haemorrhagic groups, and the inclusion number of the patient. Numbers appears clearly on packages and labels for researchers and laboratory. Unblinding envelopes are available in cases of emergency.

##### Compliance with the drug administration will be checked during the follow-up visits. All participants will be instructed to return any unused drugs to the investigator on the last follow-up visit after treatment

ᅟ

##### Methods of storing the investigational drug

The preparation of 10-mL study syringes will be achieved by the pharmacy of the sponsor in numbered boxes depending of the randomisation by centre. Every syringe and kit will be labelled according to the current regulation. The boxes are allocated to the centres via their pharmacy research unit and four boxes will be available on the labour ward in order to respond to elective or emergency inclusion processes. The product is allocated to the patient following an increasing number system. Boxes are returned to the centre pharmacy for tracing.

##### Stability study and description of the study drug production

To maintain the blind during the TRACES trial, pharmacists will be solicited to prepare ready-for-use vials containing TA (Exacyl® 0.5 g/5 mL, Sanofi-Aventis, Paris, France) at 1 g/10 mL or 0.5 g/10 mL or placebo. As the stability of TA in vials at these concentrations has not been previously studied, to our knowledge, a stability study will be performed by the toxicology laboratory of the Lille centre.

The preparation consists of adding an equal volume of 0.9% sodium chloride and TA, when diluted at 0.5 g/10 mL, and aseptically filling type I glass vials after a sterilising filtration (0.2-μm cellulose acetate filtre; Minisart®, Sartorius, France). The vials are then closed with a chlorobutyle septum.

For the stability study, 28 vials of placebo, 52 vials of TA at 0.5 g/10 mL and 52 of TA at 1 g/10 mL are prepared. Vials are stored at room temperature (below 25 °C). TA concentrations are determined using a UPLC-MS/MS method developed by the toxicology laboratory. The times of analysis are day 0, day 15, day 30, day 60, day 90, 6 months, 9 months and 1 year. For each analysis, quantification of TA is performed on three vials.

Results of the stability study are presented in Table 1.

**Table 1:** TA concentration in vials over time

Concentration of TA (mg/mL)	Day 0	Day 15	Day 30	Day 60	Day 90	6 months	9 months	1 year
Vials with TA 0.5 g/10 mL	46 [45–47]	45,67 [44–49]	50.33 [49–52]	51 [50–52]	47.33 [46–50]	57.33 [54–61]	54.66 [53–56]	51 [50–52]
Vials with TA 1 g/10 mL	96 [93–101]	103.67 [102–109]	106 [102–112]	102.33 [102–103]	97 [96–98]	109.75 [106–114]	101.33 [101–102]	99.33 [99–100]

*TA* tranexamic acid

Moreover, the presence of potential impurities, those specified in the monograph of TA in the *European Pharmacopoeia*, was checked using gas chromatography and liquid chromatography-based methods. No impurities were found in vials up to 1 year.

During the same time, a microbiological stability test was conducted by directly seeding in a culture medium according to the *European Pharmacopoeia*. Times of seeding are day 0, day 90, 6 months and 1 year. Four tubes containing a brain-heart infusion medium with a coloured indicator will be inoculated for each concentration. Half of the media are incubated at 22 °C and the remaining at 37 °C. After being incubated for 14 days, no contamination was found.

In total, the stability of TA in glass vials for up to 1 year was demonstrated.

#### Blind randomised treatment administration

The preparation of the two dose regimens and the placebo is planned for each centre by the promoter to certify the double-blind character of the trial. The products will be prepared as ready to use

The treatment is presented in a blinded 10-mL syringe containing 0, 0.5 g or 1 g TA, respectively, for each TA0 (placebo), TA½ and TA1 group. The single-dose injection will be independent of patient weight in order to allow a pharmacokinetic analysis of the weight influence. Treatment administration is performed after birth.

The time of injection onset is noted as T0. Intravenous injection will be performed with a strict control of 1-min duration and the end of injection defines T1 = T0 + 1 min, the time of the plasma peak of TA.

Rescue administration of a second dose of 1 g TA is allowed only if haemorrhage becomes severe (more than 1500 mL). If this condition cannot be respected, the patient will be excluded a posteriori. The inclusion number will be kept for the patient presenting with this deviation and the follow-up and visits will continued and analysed in the intention-to-treat analysis.

The investigator can use, at any moment and in any situation that seems necessary, the unblinding procedure by opening the envelopes attached to the treatment.

#### Assessments

**Fig. 5:**
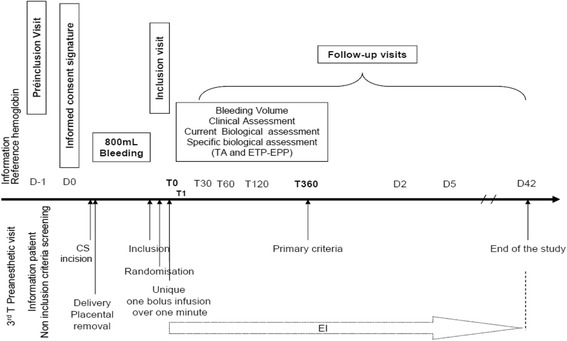
Study protocol. *CS* caesarean section, *ETP-EPP* thrombin and plasmin generation potential in a well

**Fig. 6:**
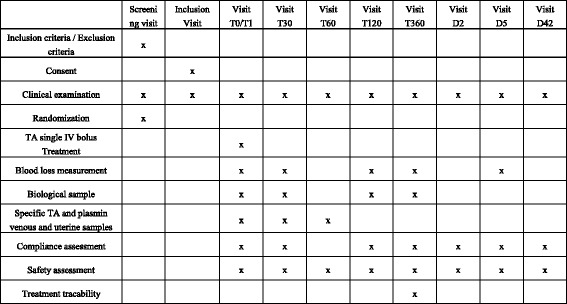
Schedule of enrolment, interventions, and assessments adapted from the Standard Protocol Items: Recommendations for Interventional Trials (SPIRIT) Figure

Assessments are described in Figs. 5 and 6 describing, respectively, the study protocol and the Standard Protocol Items: Recommendations for Interventional Trials (SPIRIT) flow chart and assessments at each repeated exploration time Additional file 1.

##### Primary endpoint: blood loss measurement method, timing and precision. The primary endpoint is the additional blood loss after injection measured at 6-h follow-up (T360)

Blood loss volume (mL) is measured in the surgical or cell-saver aspiration bag, in the delivery bag collecting vaginal blood flow during CS and by weighing drapes and pads (conversion factor for blood density = 1.06). Antiseptic and amniotic fluids must be strictly separated, counted and subtracted.

Precise blood-loss volume measurement using the physical method is guaranteed as follows: collection of the blood in a surgical or cell-saver aspiration bag is the method of measurement used in major surgery and is more precise during CS than in delivery bag collection after vaginal delivery. Drapes and pads are weighed at each observation time. Amniotic fluid, washing solutions and antiseptics are subtracted or collected in a separate aspiration bag. The investigation centres have been selected regarding their routine practice in surgical blood measurement during CS. Blood-loss calculation can be biaised by the haemodilution/haemoconcentration process and by haemolysis. However, in order to obtain a double assessment of the primary criterion, the haemoglobin-(Hb)-drop-based blood loss calculation will be performed using haemoglobin drop (last Hb measurement available before delivery as reference to day-2 final Hb) corrected by the transfused haemoglobin amount (red blood cell (RBC) concentration of 54 g/100 mL).

Secondary endpoints aim to produce scientific data for dose-ranging reduction of maternal morbidity: total blood loss, postpartum anaemia, postpartum organ failure, late postpartum side effects and death.

The pharmaco-biological substudy aims to provide data and the TA dose-ranging effect on plasmin generation and thrombin generation with regard to TA plasmatic and uterine concentration. Secondary endpoints will be measured before inclusion (T0) and at T30, T60, T120 and T360, respectively 30, 60, 120 and 360 min after inclusion and at day 2 (±12 h)

##### Safety assessments

Side effects will be recorded at each study time (Fig. 6).

High doses of TA have been implicated in the development of seizures and visual disturbances in the context of cardiac surgery. In the trauma context [27], as well as in the EXADELI trial [21], no excessive rates of complication (myocardial ischaemia, stroke, deep vein thrombosis, or renal failure) had been identified in the treated group compared to placebo. However, arterial or venous thrombotic risks must be monitored considering the prohaemostatic characteristics of the drug. Nausea and vomiting have been previously reported with TA [21].

##### Data collection

Inclusions are performed 24 h per day, 7 days a week. Each patient admitted for an elective or non-emergency CS during labour will be informed before the CS commences and a signed consent will be obtained. Patients will be included in the haemorrhagic group when haemorrhage exceeds 800 mL. Blind injection of the 10 mL-vial containing 0, 0.5 g or 1 g of TA will be performed over 1 min between T0 and T1 after injection. Transfusion, maternal haemodynamic, resuscitation measure, uterotonic and invasive procedure data and maternal morbidity parameters are recorded before inclusion (T0) and at T30, T60, T120 and T360, respectively 30, 60, 120 and 360 min after inclusion and at day 2 (±12 h) and day 42 (±14 days) postpartum. D42 data collection will be performed using telephone interview.

Various margins of time will be allowed for the timing of the data collection: T1 is the reference time and the time of the TA plasma concentration peak and no time margin will be allowed (sample exactly at the end of the TA injection). The time margins allowed for T30, T60, T120, T360 will be 5, 5, 10 and 60 min respectively. The margins allowed for day 2 will be 12 h and for day 42, 14 days. The real data collection time will be collected exactly anyway to allow the pharmacodynamic analysis.

##### Biological assessment

Preanalytical management

Tubes will be prepared in advance by the local investigator. Samples are identified by coloured codes. Samples are brought to the laboratory rapidly and current biological parameters of coagulation, blood count and renal function are performed on site and communicated directly to the clinician in charge of the patient. Concerning the TA concentration analyses and simultaneous thrombin-plasmin generation assay (STPGA), as well as any future contributive biological method to diagnose and treat fibrinolysis, plasma samples will be separated by centrifugation (2500 g × 15 min at 20 °C), then collected and frozen to be kept at − 70 °C in each centre in a box with the inclusion number and the time of sampling in order to identify the sample. At the end of the inclusions, all plasma samples will be transferred to the Biology and Pathology Centre of the Lille University Hospital in dry ice by an agreed carrier (authorised L1243-4 in the French Public Health Code).

Non-specific biological assessment

Blood for laboratory tests will be sampled using the second venous catheter currently placed as a safety procedure for CS surgery at T0 and day 2. Laboratory non-specific tests will be carried out in each centre as recommended in the French guidelines. The reference values will be collected. Laboratory non-specific tests may include complete blood count with platelet count, coagulation screening including aPTT, PT, fibrinogen, factors II and V, antithrombin, fibrin monomers and D-dimers, as well as renal function parameters. Complete blood count will be measured at the morning of day 2 after CS to obtain the comparison with the third trimester results.

Specific biological assessment

**Fig. 7:**
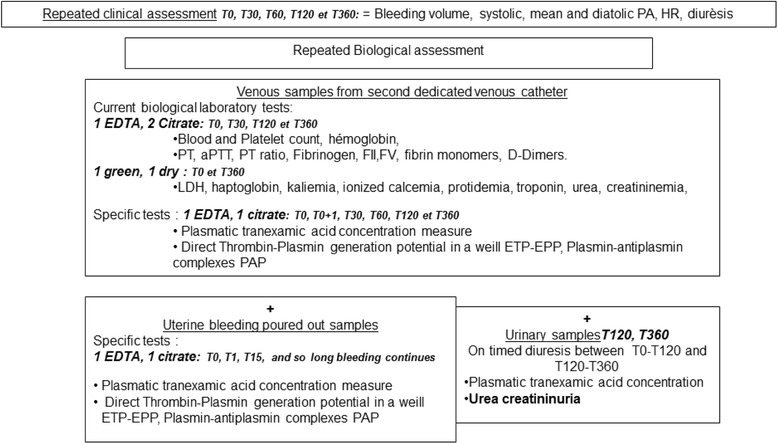
Ancillary pharmaco-biological substudy flow chart

A pharmaco-biological substudy (Fig. 7) is supported by the French National Agency for Medicine and Health Products research programme and will be described in a parallel publication. Specific venous blood samples will be performed as part of the biological sampling before inclusion and at follow-up visits at T0, T30, T60, T120 and T360. Two specific tests are planned: a STPGA in a well [28], and the determination of TA concentration [29]. Both of these innovative tests will be performed on venous blood and uterine bleeding samples in order to plot a pharmacokinetic distribution of the product and its effect on fibrinolysis. The TA concentration analysis will be performed only in the haemorrhagic-patient group. A specific sample of uterine bleeding occurring intraoperatively will be performed at T0/T1 and T15 and as long as the uterine bleeding continues. A T0–T120- and T120–T360-timed urinary sample is collected in a graduated urinary bag to measure the TA urinary cumulative excretion.

#### PPH management and care standardisation

The uterotonic treatment, vascular loading and transfusion follow the French PPH guidelines, as described in the following paragraph. Uterotonic treatment respects strictly the guidelines for the prevention of PPH: systematic prophylactic infusion of oxytocin by electric syringe (10 IU in 10 min, then 10 IU over 60 min, then 10 IU over 5 h). A 5-IU intravenous bolus and a 20-IU/20-min infusion are administered in cases of uterine atony. Sulprostone or prostaglandin treatment: 500 μg in 1 h, then 500 μg over 6 h is initiated in cases of oxytocin inefficiency.

Vascular loading uses gelatin or crystalloids to compensate for exact blood loss volume exceeding 800 mL. The criteria for colloid adjustment and haemodynamic assessment are the reduction of tachycardia to less than 100 bpm, the stability of a diuresis volume at more than 40 mL/h and a systolic blood pressure over 80 mmHg. The non-invasive haemodynamic device NEXFIN® (Edwards, Philadelphia, PA, USA) should be used for this assessment. Red blood cell transfusion is strictly conducted regarding guidelines in order to maintain the haemoglobin level at more than 8 g/dL. The follow-up of the haemoglobin level use delocalised devices as repeated HemoCue® or continuous haemoglobin monitoring (Massimo®, Meaux, France). Cell salvage is used following the international guidelines. Fibrinogen concentrates can be used in association with TA when Clauss plasma fibrinogen is less than 2 g/dL and A5 FIBTEM less than 8 mm or MCF FIBTEM less than 20 mm. Prothrombinic complex concentrates, factor XIII or recombinant factor VIIa can be discussed in cases of hysterectomy. Obstetric rescue invasive procedures follow the French guidelines for PPH management in cases of persistent massive haemorrhage: intrauterine compression balloon, embolisation or surgical arterial ligatures and surgical compression techniques. Hysterectomy is considered for maternal rescue.

### Trial conduct and data monitoring

#### Study duration

Enrolment period: 2016–2018

For a given subject: 42-day participation after randomisation

Duration of research: 44 months

End of the research term following participation of the last enrolled person: 1 year

#### Data collection and management

Data will be anomymised in accordance with the law dated 6 January 1978. Data will be collected electronically. Data will be analysed in accordance with the methodology described in MR 06001 form of the French Data Protection Authority by Prof. Alain Duhamel’s epidemiology and public health biostatistics platform Lille University Hospital Lille. Access to data will be restricted to individuals who are directly involved in the study. Data may be modified by any physician participating in the study or a fellow working with a physician participating in the study. Trial data will be archived for at least 15 years after the trial has ended.

### Study outcomes

#### Primary endpoint

The primary endpoint is the additional blood loss volume measured between inclusion (T0) and 6 h after inclusion (T360).

#### Secondary clinical endpoints

The secondary endpoints will measure the impact of the study product and dose on total blood loss, intensity and rate of postoperative anaemia (decrease of haemoglobin preoperative to day 2, nadir of haemoglobin, number of patients with haemoglobin under 8 g/dL or a 4-point decrease at any time), immediate and late red blood cell transfusion and usage of prohaemostatic products, rate of surgical interventional procedures, such as arterial ligature, or surgical compression of the uterine wall or internal compression balloon, CS duration and general anaesthesia frequency, rate of postoperative organ failure, rate and duration of intensive care hospitalisation, breast-feeding duration, death, and the safety of the dose-ranging use of TA in haemorrhagic CS, and side effects related to the product.

#### Secondary biological endpoints

The secondary biological endpoints measure the impact of the study product and dose on D-dimers level and haemostasis parameters, specifically focussed on the diagnosis and evolution of fibrinolysis, plasmin generation and plasma and uterine TA concentrations.

### Statistical method

#### Sample size calculation

The sample size computation is based on the expected difference between the placebo group and the low-dose group and available data on the TA use in active PPH.

Ducloy et al. recruited patients who experienced moderate PPH after a vaginal delivery. The treatment was a TA uniform high dose: 4 g over 1 h, then 1 g/h over 6 h. T0–T360 blood loss after inclusion was estimated at 133 ± 252 mL (mean ± standard deviation) in the placebo group and at 58 ± 96 mL in the treated arm. Kolsi et al. recruited patients who experienced moderate PPH during CS and tested a uniform TA dose of 10 mg/kg. The TA group received 10 mg/kg of TA as an induction dose within 12 min after second-line uterotonic drug onset and 1 mg/kg/h as maintenance within the two following hours. The placebo group received the same volumes of normal saline. The average of total bleeding was 1313 ± 1432 mL in the TA group vs 2089 ± 1556 mL in the placebo group (*p* = 0.03). The WOMAN trial recruited patients who experienced bleeding after vaginal delivery or CS [24]. The design of the WOMAN trial used a 1-g single TA dose. There was no volume measurement request in the study design. The recruitment concerns mostly low-resource countries and the criterion was hysterectomy and/or mortality rate. The first results led to more than 4% and 2%, respectively, of the primary criterion, 10 times higher than European morbidity rates that cannot be extrapolated.

Given that there is no available data on the impact of low TA dosage on blood-loss reduction after inclusion, and considering a maximal 50% reduction as previously observed after higher dose administration, the required number of subjects is 103 by group for a type I error of 5% and 80% power. Considering a maximum of 10% of dropouts or missing data, we will recruit 114 haemorrhagic patients by group for a total of 342 patients.

In order to compare the specific fibrinolysis inhibition parameters with the recent thrombin-plasmin potential generation in a well, a subgroup of patients will be sampled for these specific biological tests and a complementary group of non-treated, non-haemorrhagic CS patients will be included in an observational sequence of clinical and biological assessments. The number needed to observe these pharmaco-biological secondary objectives has been calculated regarding the inhibition of fibrinolysis as diagnosed by the number of patients for whom the D-dimer-level increase between 30 and 120 min was negative or null (EXADELI trial [21]). The number necessary to treat for this substudy is 48 patients in each of the three haemorrhagic groups and 48 patients in the reference non-haemorrhagic group, in total 192 patients. These substudy patients will be selected out of the 342 patients of the experimental groups and recruited during the daytime when sampling, collection and freezing are available.

#### Statistical analysis

Statistical analyses will be independently performed by the Biostatistics Department of University of Lille under the responsibility of Prof. Alain Duhamel. Data will be analysed using SAS software (SAS Institute Inc, Cary, NC, USA) and all statistical tests will be performed with a two-tailed alpha risk of 0.05. A detailed statistical analysis plan will be written and finalised prior to the database lock. Baseline characteristics will be described for each group. The quantitative variables will be expressed as mean and standard deviation in cases of normal distribution and median and interquartile if not. The normality of distributions will be checked graphically and using the Shapiro-Wilk test. Qualitative variables will be expressed as frequencies and percentages.

For the main objective, the blood loss measured in each experimental group (low-dose and high-dose) will be compared to that of the placebo group by using an analysis of covariance adjusted for baseline blood loss volume and centre effect. In cases of non-normal distribution (except if a log-transformation could be applied to normalise the data), relative blood loss volume will be calculated and compared using a Mann-Whitney *U* test.

Analyses will be done on an intention-to-treat basis especially concerning the patients receiving a rescue dose of TA in cases of severe (>1500 mL) haemorrhage. An exploratory analysis will be performed on all the measures of the primary endpoint (T0, T30, T60, T120 and T360). We will use the linear mixed model in order to compare the evolution of the primary endpoint according to each experimental group and the placebo group. This model allows the performing of an analysis of variance (ANOVA) test for repeated measures taking into account the correlation between the repeated measures. The choice of the covariance model will be based on the AIC criteria. Post hoc analysis at each time of measure will be performed using a Bonferroni correction.

For the secondary objectives, secondary endpoints will be compared between each experimental group and the placebo group by using the chi-square test or Fisher’s exact test for qualitative parameters, and using Student’s *t* test or the Mann-Whitney *U* test for quantitative parameters. For the quantitative parameters measured at each time, we will employ the linear mixed model, as previously described for the main objective. The incidence of adverse events will be analysed in a descriptive way.

### Study organisation

The coordinating team includes the coordinator, the promotor and the major investigators of each of the centres. A call conference will be organised every 2 months between the coordinator and the centres, supported by a newsletter reporting inclusion rates and protocol information. Data and consent collection will be monitored by the promotor. An independent safety monitoring committee is recruited to observe blinding safety issues and allow trial continuation. Data management and statistical analysis will be done by an independent unit. Plans are written in advance for investigators and sponsor to communicate trial results to participants, healthcare professionals, the public, and other relevant groups (e.g. via publication, reporting in results databases, or other data-sharing arrangements).

### Trial status

TRACES inclusions have already begun. In the first opened centre (promoter centre), 64 patients have been included (57 haemorrhagic and seven non-haemorrhagic). The four other centres are opened and included 13 patients. Three Safety and Monitoring Committee meetings monitored these first data and allowed the continuation of the trial.

## Additional file

Additional file 1: Trial publication spirit check list. (DOC 130 kb)

## Abbreviations

TA: Tranexamic acid
PPH: Postpartum haemorrhage
CS: Caesarean section
aPTT: PT, Activated partial prothrombin time, prothrombin time
STPGA: Simultaneous Thrombin-Plasmin Generation Assay
DIC: Disseminated intravascular coagulopathy
RBC: Red blood cells
Hb: Haemoglobin
CPP: Ethics Committee
e-CRF: Electronic Case Report Form
PHRC-N: National Health Clinical Research Programme
ANSM: French National Agency for Medicine and Health Products
